# Renal function markers and insulin sensitivity after 3 years in a healthy cohort, the EGIR-RISC study

**DOI:** 10.1186/s12882-018-0918-1

**Published:** 2018-05-31

**Authors:** Soline Siméon, Ziad Massy, Kurt Højlund, Katarina Lalic, Francesca Porcellati, Jacqueline Dekker, John Petrie, Gemma Currie, Beverley Balkau

**Affiliations:** 1 CESP team5, Faculty of Medicine - University Paris-South, Faculty of Medicine - University Versailles-St Quentin, INSERM U1018, University Paris-Saclay, Villejuif, France; 2 Division of Nephrology, Ambroise Paré Hospital APHP (Z.M.), Boulogne-Billancourt, Paris, France; 3 Department of Endocrinology (K.H.) Odense University Hospital, DK-5000 Odense, Denmark; 4 The Section of Molecular Diabetes & Metabolism, Department of Clinical Research and Institute of Molecular Medicine, University of Southern Denmark, DK-5000 Odense, Denmark; 5 Faculty of Medicine, University of Belgrade, Clinic for Endocrinology, Diabetes and Metabolic Diseases, Belgrade, Serbia; 6 Section of Internal Medicine, Endocrinology and Metabolism, Department of Medicine, Perugia University School of Medicine, Perugia, Italy; 7 Department of Epidemiology and Biostatistics, Amsterdam Public Health research institute, VU University Medical Center, Amsterdam, the Netherlands; 8 Institute of Cardiovascular and Medical Sciences, University of Glasgow, Glasgow, Scotland, UK; 9 CESP, INSERM U1018 Equipe 5, 16 Avenue Paul Vaillant Couturier, 94807 Villejuif cedex, France

**Keywords:** Albuminuria, Cohort, Epidemiology, Glomerular filtration rate, Insulin sensitivity, Renal function, Sex

## Abstract

**Background:**

People with chronic renal disease are insulin resistant. We hypothesized that in a healthy population, baseline renal function is associated with insulin sensitivity three years later.

**Methods:**

We studied 405 men and 528 women from the European Group for the study of Insulin Resistance - Relationship between Insulin Sensitivity and Cardiovascular disease cohort. Renal function was characterized by the estimated glomerular filtration rate (eGFR) and by the urinary albumin-creatinine ratio (UACR). At baseline only, insulin sensitivity was quantified using a hyperinsulinaemic-euglycaemic clamp; at baseline and three years, we used surrogate measures: the Matsuda insulin sensitivity index (ISI), the HOmeostasis Model Assessment of Insulin Sensitivity (HOMA-IS). Associations between renal function and insulin sensitivity were studied cross-sectionally and longitudinally.

**Results:**

In men at baseline, no associations were seen with eGFR, but there was some evidence of a positive association with UACR. In women, all insulin sensitivity indices showed the same negative trend across eGFR classes, albeit not always statistically significant; for UACR, women with values above the limit of detection, had higher clamp measured insulin sensitivity than other women. After three years, in men only, ISI and HOMA-IS showed a U-shaped relation with baseline eGFR; women with eGFR> 105 ml/min/1.73m^2^ had a significantly higher insulin sensitivity than the reference group (eGFR: 90–105 ml/min/1.73m^2^). For both men and women, year-3 insulin sensitivity was higher in those with higher baseline UACR. All associations were attenuated after adjusting on significant covariates.

**Conclusions:**

There was no evidence to support our hypothesis that markers of poorer renal function are associated with declining insulin sensitivity in our healthy population.

**Electronic supplementary material:**

The online version of this article (10.1186/s12882-018-0918-1) contains supplementary material, which is available to authorized users.

## Background

Many studies have investigated the relation between chronic kidney disease (CKD) and insulin sensitivity, but it is still not clear whether reduced insulin sensitivity precedes CKD or the inverse. Most of the epidemiological studies are cross-sectional, so they cannot resolve this issue, and the few prospective studies have approached the question from the hypothesis that low insulin sensitivity precedes, or perhaps causes, the decline in kidney function.

Early clinical studies focused on insulin sensitivity in people with CKD and used labor intensive methods such as the hyperinsulinemic-euglycaemic clamp, the reference method, to measure insulin sensitivity. De Fronzo et al. reported in a study of 17 people with chronic uremia but without diabetes, and 36 controls, that peripheral insulin resistance was the primary cause of insulin resistance, not hepatic insulin resistance [1]. Fliser et al. investigated insulin sensitivity by the frequently sampled intravenous glucose tolerance test in people at various stages of renal disease [2]. In this small study of 50 people, there was a trend for lower insulin sensitivity in the group with the highest plasma creatinine levels.

There are a number of large cross–sectional epidemiological studies on insulin resistance with either estimated glomerular filtration rate (eGFR) and/or CKD [3–12]. Most found a relation between either eGFR or the prevalence of CKD with insulin resistance measured by the HOmeostasis Model Assessment of Insulin Resistance (HOMA-IR) [13] or by insulin concentrations, but not always after adjustment for confounders [5, 10, 11].

There are prospective studies that investigate whether low insulin sensitivity precedes CKD or a decline in eGFR [8, 14–17]. Some show that a lower insulin sensitivity is associated with progression to incident CKD [15–17], one shows a decline in eGFR but no relation with incident CKD [8], and Fox et al. do not show any statistically significant relation [14]. An article from the EGIR-RISC cohort [18] reports that a higher baseline insulin sensitivity is associated with a lower urinary albumin creatinine ratio (UACR) at 3 years [19].

The possibility that renal function causes a lowering in insulin sensitivity needs to be explored. Further, whether associations differ between men and women is rarely investigated. Cardiovascular risk factors and insulin sensitivity differ between men and women in the European Group for the study of Insulin Resistance - Relationship between Insulin Sensitivity and Cardiovascular disease (EGIR-RISC) cohort [18] with sex-specific relations between insulin sensitivity and intima-media thickness parameters, blood pressure and sleep characteristics [20–22].

We investigate in a healthy population, whether renal function, as measured by eGFR and UACR, is associated with insulin sensitivity after three years of follow-up.

## Methods

The cohort EGIR-RISC aimed to evaluate whether insulin resistance is involved in the development of cardiovascular diseases, in a population without diabetes, hypertension, renal disease or dyslipidaemia [18]. The study was approved by ethics committees in each recruitment centre, the declaration of Helsinki was adhered to and participants gave written informed consent to participate in the study.

The 1259 men and women included in the study were aged 30 to 60 years (flow chart of inclusions Additional file 1: Figure S1). Criteria for non-inclusion were renal disease (participants responded to the question whether they had ‘kidney failure, kidney dialysis or transplant’ and from study results of eGFR and UACR), diabetes (treated or from the results of an oral glucose tolerance test (OGTT)), hypertension, dyslipidaemia or treatment for any of these pathologies [18].

The population for the present study included 405 men and 528 women who had measures of creatinine and a clamp measure of insulin sensitivity at baseline. In this healthy population, none of the individuals had macroalbuminuria at inclusion (UACR ≥300 mg/mmol) and the lowest eGFR was 59 ml/min/1.73 m^2^.

### At baseline and 3 years

Participants completed questionnaires detailing smoking habits, alcohol intake, physical activity, and weight, height, blood pressures, heart rate were measured. They underwent a 120 min OGTT, with blood drawn every 30 min, for assays of glucose and insulin. Plasma and serum samples were frozen at − 80 °C and all assays were centralized [18].

The glomerular filtration rate was estimated using the Chronic Kidney Disease EPIdemiology collaboration equation (CKD-EPI) [23]. This was analysed as a continuous variable and in three classes eGFR < 90; 90–105; > 105 ml/min/1.73m^2^; the threshold 90 ml/min/1.73m^2^ was chosen as it is conventionally used to indicate kidney disease without chronic kidney failure [23]; the group with eGFR > 105 ml/min/1.73m^2^ includes half of our healthy population. At baseline, the UACR was determined on two separate occasions several weeks apart, and the mean UACR calculated and analysed in three classes: undetected, detected below and detected above the sex-specific median.

The reference method of measuring insulin sensitivity, the hyperinsulinaemic-euglycaemic clamp [1, 18, 24] was used at baseline. This involved an infusion of insulin at the rate of 240 pmol/min/m^2^ and every 5 to 10 min, the glucose infusion rate was adjusted so that the concentration remained within 0.8 mmol/l of the target glucose concentration, set between 4.5 and 5.5 mmol/l [18]. M/I quantified insulin sensitivity, where M is the glucose infusion rate and I the insulin concentration over the last 40 min of the 2-h clamp. The two surrogate measures of insulin sensitivity were the Matsuda Insulin Sensitivity Index (ISI) [25]:

$$ {\displaystyle \begin{array}{l}\mathrm{ISI}=10,000/\left\{\surd \right[\ \left(\ \mathrm{fasting}\ \mathrm{plasma}\ \mathrm{insulin}\ \right)\ \mathrm{x}\ \left(\ \mathrm{fasting}\ \mathrm{plasma}\ \mathrm{insulin}\ \right)\\ {}\kern12em \mathrm{x}\ \left(\ \mathrm{mean}\ \mathrm{OGTT}\ \mathrm{glucose}\ \mathrm{concentration}\Big)\ \mathrm{x}\ \left(\ \mathrm{mean}\ \mathrm{OGTT}\ \mathrm{insulin}\ \mathrm{concentration}\ \right)\ \right]\end{array}} $$

and the HOmeostasis Model Assessment of Insulin Sensitivity (HOMA-IS = 1/HOMA-IR) [13]:

$$ \mathrm{HOMA}-\mathrm{IR}=\left(\ \mathrm{fasting}\ \mathrm{plasma}\ \mathrm{insulin}\ \right)\ \mathrm{x}\ \left(\ \mathrm{fasting}\ \mathrm{plasma}\ \mathrm{insulin}\ \right)/22.5. $$

For prospective analyses, the yearly changes in these two parameters were calculated.

Glucose and insulin were assayed at the Odense University hospital in Denmark, by respectively, glucose oxydase and immunofluorescence techniques.

Creatinine was assayed from frozen samples in the Centre for Cardiovascular Research in Glasgow, United Kingdom, using an enzymatic isotope dilution mass spectrometry standardized method.

Urinary albumin and creatinine were assayed in Amsterdam at baseline and at 3 years, using a Beckmen array 360 protein analyser, and a Jaffe creatinine reagent on a modular P system (Roche).

The lipid profile was from the biochemistry laboratory in Dublin, Ireland. Total cholesterol, HDL-cholesterol and triglycerides were assayed by enzymatic colorimetric techniques (Roche cholesterol method for modular systems, Roche HDL 2^nd^Genmethod for modular systems and Roche Triglycerides method for modular systems respectively); LDL-cholesterol was calculated from the Friedwald formula. Leptin and adiponectin were assayed respectively, by the immunologic DELFIA® method in the department of clinical biochemistry in Cambridge, UK and by immunoflurorescence in the biochemical laboratory, University of Aarhus, Denmark. Liver enzymes were assayed by the Bergmeyeres method, according to the International Federation of Clinical Chemistry recommendations for alanine aminotransferase (ALAT) and aspartate aminotransferase (ASAT) and by an enzymatic colorimetric method for gamma glutamyltransferase (GGT) in Glasgow; at baseline only, interleukin-6 (IL-6) and 25 hydroxy vitamin D were also assayed in Glasgow, by respectively, an ELISA method and by the ‘competitive principle’ on a Roche/Hitachi Cobas c 311 (Burgess Hill).

### Statistical analysis

Characteristics of participants are presented as the median (first and third quartiles) or as n (percentage). Kruskal-Wallis and χ^2^ tests compared the characteristics of those included and not included in the study, and also compared men and women. At baseline, Spearman partial correlation coefficients quantified the association between the three measures of insulin sensitivity, adjusted on age and the recruitment centre. The insulin sensitivity parameters (M/I, ISI and HOMA-IS) were log-transformed before analysis.

All analyses were stratified on sex, as the regression analysis of ln(M/I) on eGFR showed a trend for a sex interaction (*P*_*inter*_ = 0.068).

Cross-sectional associations of insulin sensitivity as dependent variables, with renal function indicators (eGFR as a continuous variable and in classes, UACR in classes) and potential covariates were studied, one-by-one, using general estimating equation methods, adjusted on age and recruitment centre, as a random factor. Fractional polynomial transformations were allowed if statistically significant [26, 27] and multivariable results used a backwards stepwise selection procedure to select variables associated with insulin sensitivity. Only *linear* functions of eGFR were chosen by the fractional polynomial transformation procedure.

The relation between renal function markers at baseline with ISI and the HOMA-IS indices at year-3, and their yearly changes, were studied with similar methods.

Analyses used SAS version 9.3 and STATA version 12. Statistical tests were two-sided and *P* < 0.05 was considered statistically significant; we have used a more liberal value for the interaction as interaction tests are known to be lacking in power.

## Results

### Baseline characteristics

The 933 participants studied were older (median 44 vs 41 years) than the 326 not-studied, with a more healthy profile, a better insulin sensitivity on all three indices, but no differences for eGFR or UACR (Additional file 1: Table S1). The median eGFR was 106 ml/min/1.73m^2^ and only one person had an eGFR under 60 ml/min/1.73m^2^, three people had an eGFR > 150 ml/min/1.73m^2^; 18 individuals (2%) had microalbuminuria, and none macroalbuminuria.

Most characteristics differed between men and women (Table 1) with a worse profile in the men. An exception was eGFR where there was no sex-difference, but the mean UACR was lower in men than women.

**Table 1:** Characteristics [median (quartile 1- quartile3) or n (%)] of the study population at inclusion, by sex. The *P*-values are from Kruskal Wallis or χ^2^ tests. The EGIR-RISC Study

Characteristics	Men (*n* = 405)	Women (*n* = 528)	*P*-value
Age (years)	43 (37–51)	45 (39–50)	0.082
Current smoker	108 (27%)	131 (25%)	0.52
Alcohol (g/week)	79 (39–153)	35 (11–68)	<.0001
Physical activity (met-mins/week)	2331 (1040–4599)	2165 (1032–4958)	0.92
BMI (kg/m^2^)	25.7 (23.8–27.9)	23.9 (21.9–26.8)	<.0001
Systolic blood pressure (mmHg)	122 (115–130)	114 (104–122)	<.0001
Heart rate (bpm)	64 (58–72)	70 (63–77)	<.0001
Renal function parameters			
Creatinine (μmol/l)	75 (67–83)	59 (51–66)	<.0001
Estimated glomerulaire filtration rate (ml/min/1.73 m^2^)	106 (98–114)	106 (96–114)	0.53
< 90	61 (15%)	78 (15%)	
90 to 104.9	120 (29%)	167 (31%)	0.81
≥ 105	224 (56%)	283 (54%)	
Urinary albumin-creatinine ratio (mg/mmol)	0.18 (0–0.35)	*n* = 520 0.26 (0–0.47)	0.001
not detected	104 (26%)	139 (27%)	
detected but < 0.26/0.36 men/women	148 (36%)	187 (36%)	0.94
≥ 0.26/0.36 men/women	153 (38%)	194 (37%)	
Insulin sensitivity indices			
M/I (μmol/min/Kg.ffm/nM)	114 (85–154)	147 (112–192)	<.0001
ISI	9.0 (6.5–12.8)	9.6 (6.6–13.9)	0.038
HOMA-IS	14 (10–20)	16 (11–24)	0.0036
Biological characteristics			
Fasting glucose (mmol/l)	5.2 (4.9–5.5)	5.0 (4.7–5.3)	<.0001
2 h glucose (mmol/l)	5.5 (4.6–6.5)	5.6 (4.7–6.7)	0.033
Fasting insulin (pmol/l)	30 (22–44)	29 (20–40)	0.060
2 h insulin (pmol/l)	124 (73–216)	152 (102–242)	<.0001
Total cholesterol (mmol/l)	4.9 (4.3–5.4)	4.8 (4.2–5.3)	0.065
LDL-cholesterol (mmol/l)	3.0 (2.6–3.6)	2.8 (2.3–3.3)	<.0001
HDL-cholesterol (mmol/l)	1.2 (1.1–1.4)	1.6 (1.3–1.8)	<.0001
Triglycerides (mmol/l)	1.1 (0.8–1.5)	0.8 (0.6–1.1)	<.0001
Adiponectin (mg/l)	6.2 (4.7–7.8)	9.3 (7.2–12.2)	<.0001
Leptin (ng/ml)	4.6 (2.2–7.5)	14.6 (9.4–24.4)	<.0001
Alanine aminotransferase (IU/l)	16 (12–22)	11 (8–15)	<.0001
Aspartate aminotransferase (IU/l)	22 (17–27)	18 (14–23)	<.0001
Gamma glutamyltransferase (IU/l)	19 (13–29)	12 (8–17)	<.0001
Interleukin-6 (pg/ml)	0.74 (0.50–1.17)	0.68 (0.49–1.12)	0.12
25-OH vitamin D (ng/ml)	21 (14–28)	19 (12–27)	0.0079

The two surrogate indices of insulin sensitivity were correlated with the clamp measure (M/I), with Spearman correlation coefficients, adjusted for age and recruitment centre of 0.62 and 0.60 for the Matsuda Insulin Sensitivity Index (ISI), in men and women respectively and 0.50 and 0.49 for HOMA-IS (Additional file 1: Table S2).

### Cross–sectional analyses

**Fig. 1:**
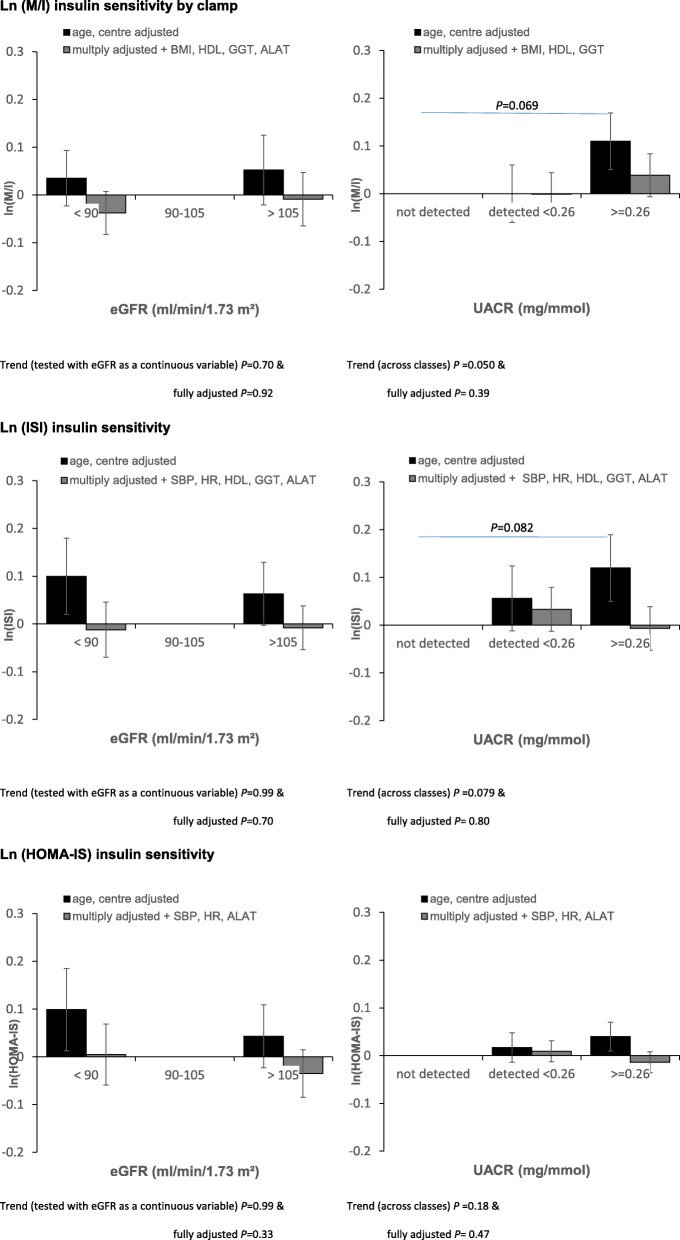
Differences in mean values (standard errors) of baseline insulin sensitivity according to baseline renal function markers in men from the EGIR-RISC Study with reference groups (90–105 ml/min/1.73m^2^) for eGFR, not detected for UACR), adjusted for age and recruitment centre, and then multiply adjusted for significant covariates: triglycerides, adiponectin, leptin and for the other parameters shown in the six individual figures. *P*-values are shown comparing groups when *P* < 0.1: full lines for age and centre adjusted and dotted lines for multiple adjustment

For men, there was no relation between eGFR and insulin sensitivity. However, over the three UACR classes there was a statistically significant linear trend for M/I, with high UACR being associated with higher insulin sensitivity (*P* = 0.050) (Fig. 1); similar but non-significant relations were seen for ISI and HOMA-IS. A number of variables were related with the three insulin sensitivity indices, notably physical activity, body mass index (BMI), heart rate, lipids, adiponectin, leptin, transaminases, IL-6, vitamin D (Additional file 1: Table S3). After adjusting for significant covariates, none of the relations between renal function markers and insulin sensitivity indices approached statistical significance. eGFR and UACR had a low Spearman correlation coefficient of 0.018, and when both were included in a multivariable regression equation, neither was significant and there was no interaction.

**Fig. 2:**
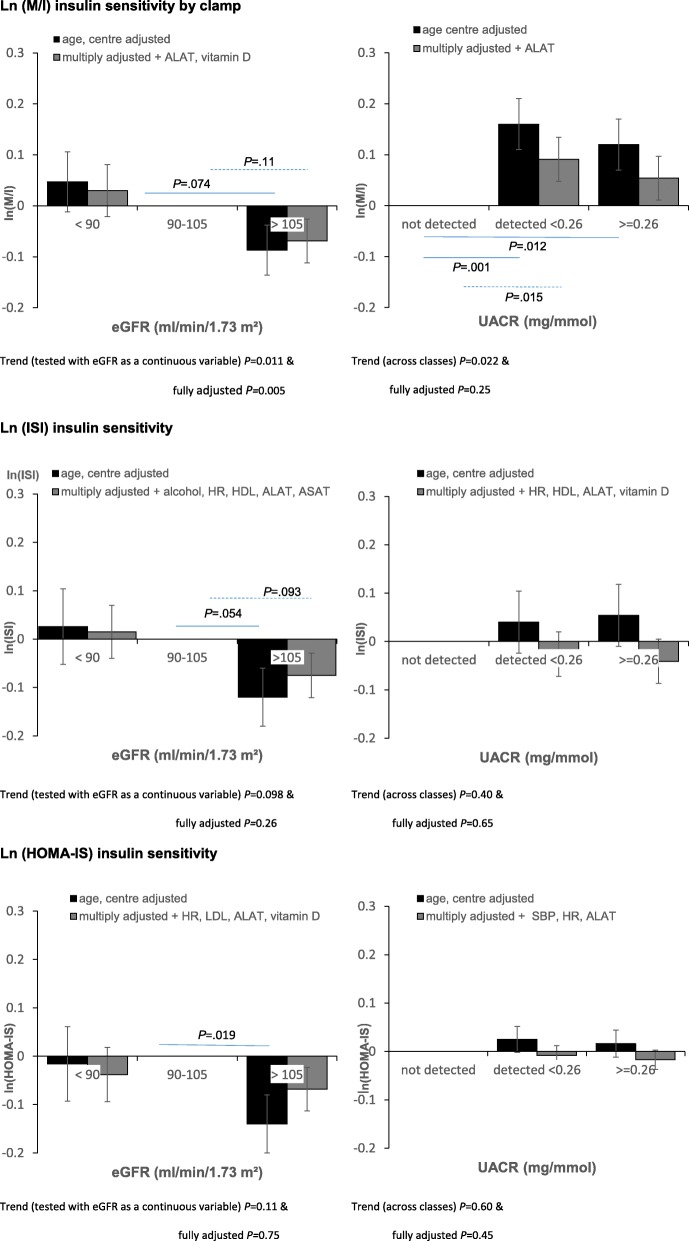
Differences in mean values (standard errors) of baseline insulin sensitivity according to baseline renal function markers in women from the EGIR-RISC Study, with reference groups (90–105 ml/min/1.73m^2^) for eGFR, not-detected for UACR), adjusted by age and recruitment centre, and then multiply adjusted for significant covariates: triglycerides, adiponectin, leptin and for the other parameters shown in the six individual figures. *P*-values are shown comparing groups when *P* < 0.10: full lines for age, centre adjusted and dotted lines for multiple adjustment

For women, eGFR was associated with clamp based insulin sensitivity – the lower the eGFR, the higher the insulin sensitivity (Fig. 2), a linear association that remained after adjusting for covariates (*P* = 0.005). For the two other insulin sensitivity indices, there was a tendency for a trend, but after further adjustment these relations were attenuated. Women with a detectable UACR had a higher M/I than women with a low non-detected UACR, but this association was attenuated after multiple adjustment. The two other insulin sensitivity indices were not associated with UACR.

Thus, for men there was no association between eGFR and clamp measured insulin sensitivity, for women there was a statistically significant inverse association.

### Prospective analyses, does renal function predict insulin sensitivity?

Over the three years of follow up, the two measures of insulin sensitivity decreased, eGFR decreased, UACR increased (Additional file 1: Table S4), with no differences between men and women.

For men, at three years both insulin sensitivity indices showed a U shaped relation with baseline eGFR: high and low eGFR groups had higher insulin sensitivity than the reference group; this relation remained for the 3-year HOMA-IS, after adjustment for covariates (Table 2). The yearly change, ΔISI, was higher for baseline eGFR> 105 ml/min/1.73m^2^ in comparison with the reference group, and this remained statistically significant after adjustment. Higher levels of baseline UACR were associated with a better insulin sensitivity at year-3, with HOMA-IS showing a stronger association that remained significant after adjustment for confounders. There was little evidence of association with the changes in insulin sensitivity and baseline UACR.

**Table 2:** Differences (95% confidence intervals) in 3-year insulin sensitivity indices (ISI and HOMA-IS) and their 3-year changes (ΔISI, ΔHOMA-IS) associated with one unit or class increase in baseline renal function parameters (estimated glomerular filtration rate (eGFR), urinary creatinine ratio (UACR)) Model 1: adjusted for recruitment centre (random effect) and age; Model 2: additionally adjusted for significant baseline variables, as indicated in the footnote^a^. The EGIR-RISC Study - Men

	ln (ISI year 3)		Ln (HOMA-IS year 3)		ΔISI		ΔHOMA-IS	
	difference (95% CI)	*P*	difference (95% CI)	*P*	difference (95% CI)	*P*	difference (95% CI)	*P*
eGFR (ml/min/1.73 m^2^)	*n* = 376		*n* = 392		*n* = 349		*n* = 380	
Model 1	trend	0.34	trend	0.35	trend	0.11	trend	0.47
eGFR> 105	.15 (.01, .30)	0.041	.093 (.030,.157)	0.004	1.5 (.2, 2.8)	0.015	2.1 (−1.2, 5.4)	0.21
eGFR 90–105	0		0		0		0	
eGFR < 90	.14 (−.05, .33)	0.13	.094 (.014, .173)	0.021	.65 (−.90, 2.20)	0.41	1.7 (−2.6,6.0)	0.43
Model 2^a^	trend	0.44	trend	0.66	trend	0.11	trend	0.21
eGFR > 105	.10 (−.01,.21)	0.069	.064 (.014, .115)	0.011	1.6 (.4, 2.9)	0.010	3.2 (−.1,6.5)	0.053
eGFR 90–105	0		0		0		0	
eGFR < 90	.038 (−.097, .174)	0.58	.058 (−.004, .121)	0.064	.75 (−.80, 2.30)	0.34	1.6 (−2.6, 5.7)	0.45
UACR (mg/mmol)	*n* = 376		*n* = 392		*n* = 349		*n* = 380	
Model 1	trend	0.026	trend	0.001	trend	0.49	trend	0.22
UACR not detected	0		0		0		0	
UACR detected but < 0.26	−.002 (−.153, .149)	0.98	.032 (−.033, .098)	0.33	−.57 (−1.84, 0.72)	0.39	−.26 (−3.72, 3.21)	0.88
UACR ≥ 0.26	.16 (.00, .31)	0.039	.10 (.03,.17)	0.002	.34 (−.92, 1.62)	0.59	1.9 (−1.4, 5.4)	0.26
Model 2^a^	trend	0.38	trend	0.009	trend	0.38	trend	0.050
UACR not detected	0		0		0		0	
UACR detected but < 0.26	−.027 (−.139, .084)	0.63	.026 (−.024, .077)	0.31	−.51 (−1.78, .78)	0.44	.66 (−2.70,4.03)	0.70
UACR≥ 0.26	.042 (−.068, .152)	0.45	.064 (.014, .114)	0.011	.47 (−.80,1.74)	0.47	3.2 (−.1,6.5)	0.062

^a^Multivariable models adjusted on age and recruitment centre, and on other significant covariates:

ln (ISI year3) and eGFR, adjusted on: HDL-cholesterol, trigylcerides, adiponectin, leptin ALAT, Il-6

ln (ISI year3) and UACR, adjusted on: HDL-cholesterol, trigylcerides, adiponectin, leptin ALAT

ln (HOMA-IS year 3) and eGFR, adjusted on: alcohol consumption, triglycerides, adiponectin, leptin, ALAT, IL-6

ln (HOMA-IS year 3) and UACR, adjusted on: alcohol consumption, heart rate, HDL-cholester ol, triglycerides, adiponectin, leptin, ALAT

ΔISI and eGFR, UACR, adjusted on: leptin

ΔHOMA-IS and eGFR, UACR, adjusted on: alcohol consumption, GGT

**Table 3:** Differences (95% confidence intervals) in 3-year insulin sensitivity indices (ISI and HOMA-IS) and their 3-year changes (ΔISI, ΔHOMA-IS) associated with one unit or class increase in baseline renal function parameters (estimated glomerular filtration rate (eGFR), urinary creatinine ratio (UACR)) Model 1: adjusted for recruitment centre (random effect) and age; Model 2: additionally adjusted for significant baseline variables, as indicated in the footnote^a^. The EGIR-RISC Study - Women

	ln (ISI year 3)		ln (HOMA-IS year 3)		ΔISI		ΔHOMA-IS	
	difference (95% CI)	*P*	difference (95% CI)	*P*	difference (95% CI)	*P*	difference (95% CI)	*P*
eGFR (ml/min/1.73 m^2^)	*n* = 492		*n* = 514		*n* = 448		*n* = 501	
Model 1	trend	0.050	trend	0.15	trend	0.46	trend	0.40
eGFR > 105	−.086 (−.200, .028)	0.14	−.027 (−.150, .096)	0.67	.81 (−.35, 1.97)	0.17	2.5 (0.2, 4.8)	0.032
eGFR 90–105	0		0		0		0	
eGFR < 90	.050 (−.101, .201)	0.51	.072 (−.084, .228)	0.37	1.1 (−.4, 2.6)	0.12	.74 (−2.10, 3.58)	0.61
Model 2^a^	trend	0.29	trend	0.55	trend	0.55	trend	0.55
eGFR > 105	−.050 (−.146, .047)	0.31	−.010 (.-.11, .09)	0.84	.76 (−.37, 1.91)	0.19	2.19 (−0.06, 4.44)	0.057
eGFR 90–105	0		0		0		0	
eGFR < 90	.011 (−.108, .131)	0.86	.016 (−.110, .141)	0.81	1.02 (−.37, 2.42)	0.15	.75 (−2.06, 3.57)	0.60
UACR (mg/mol)	*n* = 486		*n* = 507		*n* = 442		*n* = 494	
Model 1	trend	0.009	trend	0.023	trend	0.063	trend	0.004
UACR not detected	ref		ref		ref		ref	
UACR detected but < 0.36	.11 (−.01, .24)	0.080	.11 (−.02, .24)	0.10	.57 (−.60, 1.75)	0.34	.69 (−1.66, 3.03)	0.56
UACR≥ 0.36	.16 (.04, .29)	0.008	.15 (.02 .28)	0.021	1.11 (−0.06, 2.29)	0.064	3.33 (.99, 5.698)	0.005
Model 2^a^	trend	0.029	trend	0.21	trend	0.044	trend	0.007
UACR not detected	ref		ref		ref		ref	
UACR detected but < 0.36	.044 (−.056, .145)	0.38	.022 (−.080, .125)	0.67	.69 (−.48, 1.85)	0.25	.56 (−1.77, 2.88)	0.64
UACR ≥ 0.36	.11 (.008, .21)	0.032	.063 (−.038, .164)	0.22	1.20 (.03, 2.37)	0.044	3.1 (0.7, 5.4)	0.010

^a^Multivariable models adjusted on age and recruitment centre, and on other significant covariates:

Ln(ISI year 3) and eGFR, adjusted on: current smoker, BMI, heart rate, HDL cholesterol, triglycerides, adiponectin, leptin, GGT

Ln(ISI year 3) and UACR, adjusted on: current smoker, BMI, heart rate, HDL cholesterol, triglycerides, adiponectin, leptin

Ln(HOMA-IS year 3) and eGFR adjusted on: physical activity, BMI, heart rate, HDL-cholesterol, triglycerides, adiponectin, leptin

Ln(HOMA-IS year 3) and UACR adjusted on: alcohol consumption, physical activity, BMI, heart rate, HDL-cholesterol, triglycerides,

adiponectin, leptin

ΔISI and eGFR, UACR, adjusted on: LDL-cholesterol, ALAT

ΔHOMA-IS and eGFR, UACR, adjusted on: LDL-cholesterol

For women, baseline eGFR was linearly associated with 3-year ISI, the lower eGFR, the higher ISI, similar to the cross-sectional results, but this lost statistical significance after adjustment (Table 3). A higher baseline eGFR was associated with a greater positive ΔHOMA-IS, and this association was a little attenuated after adjustment. A higher baseline UACR was associated with higher 3-year ISI and HOMA-IS, and higher increases in both ΔISI and ΔHOMA-IR, and most of these relations remained significant after adjustment.

## Discussion

Data from the EGIR-RISC study did not confirm our hypothesis, that worsening markers of renal function (eGFR and UACR) precede declining insulin sensitivity in a healthy population, with renal markers within the normal range. In fact, we observed associations between a higher insulin sensitivity and markers of a worsening renal function.

In cross-sectional analyses, the insulin sensitivity indices were not related with markers of renal function in men, except for higher insulin sensitivity in those with higher UACR; this was no longer significant after adjustment for covariates. In women, clamp measured insulin sensitivity was higher in those with a lower eGFR, and this linear association remained after adjustment; clamp measured insulin sensitivity was also higher in women with detectable UACR, but this relation was attenuated after adjustment.

In longitudinal analyses, men with a higher and a lower eGFR had a higher insulin sensitivity at year-3 than the reference group (eGFR 90–105 ml/min/1.73^2^). In agreement, larger increases in ΔISI and ΔHOMA-IS were also related with a low eGFR. These relations remained consistent after adjustment for covariates. The higher the baseline UACR, the higher the year-3 insulin sensitivity. For women, there were no such relations between baseline eGFR and insulin sensitivity indices, but as for men, the higher the baseline UACR the higher the 3-year insulin sensitivity.

In the literature, the relation between insulin resistance and renal function has mainly been studied in people with renal disease, who were compared to people without renal disease. Cross-sectional studies have reported that insulin resistance exists in people without diabetes but with chronic kidney disease. DeFronzo showed that peripheral insulin resistance is present in people with chronic renal failure, but hepatic insulin resistance may not be impaired [1]. Peripheral insulin sensitivity is essentially insulin sensitivity in skeletal muscle [28]. HOMA-IS is based on fasting glucose and fasting insulin, and reflects hepatic insulin sensitivity, but could also reflect insulin clearance. ISI uses insulin and glucose levels during the two-hour oral glucose tolerance test, providing a dynamic estimate, reflecting both hepatic and peripheral insulin sensitivity [29]. The correlations between the clamp based insulin sensitivity measure and the surrogate indices were 0.50–0.60, similar to other studies.

The earliest publication in a population without diabetes or renal disease comes from Japan; a significant positive association was seen between insulin levels and serum creatinine [3]. In the American NHANES III study, Chen et al. described a higher prevalence of chronic renal disease (eGFR< 60 ml/min/1.73m^2^) in people without diabetes but with a low insulin sensitivity (odds ratio 2.6 for HOMA-IR above the upper quartile in comparison with below the lower quartile) [4]. Another analysis from the same population showed that HOMA-IR was related with eGFR in men, but not in women [5]. In older populations with lower eGFR, associations were seen with insulin resistance [6, 8, 9], whereas no association was seen in a healthy population [7]. In a study of a Korean population, Park et al. conclude that “there were no meaningful differences in HOMA-IR according to eGFR group” [11]. The results from these cross-sectional studies are not consistent, and this may be due to the age of the study populations, the numbers with low eGFR, the population studied (with or without diabetes, the metabolic syndrome [12], according to BMI [10], the level of eGFR used to define chronic kidney disease) as well as the covariates used for adjustment. Our EGIR-RISC cohort is younger, only 15% had an eGFR < 90 ml/min/1.73m^2^ and hypertension was an exclusion criterion.

Some studies have evaluated prospectively, whether a lowering of insulin sensitivity precedes a decline in renal function, but not the reverse relation, that renal decline comes first. Nerpin et al. investigated the association between insulin sensitivity measured by the hyperinsulinemic-euglycemic clamp and eGFR based on cystatin-C, in a cohort of Swedish men, average age 71 years [16]. They show that a higher insulin sensitivity at baseline is associated with a lower risk of impaired renal dysfunction (eGFR< 50 ml/min/1.73m^2^) over the 7 years of the study, independently of other aspects of glucose metabolism. In the EGIR-RISC cohort, we have shown that a low baseline clamp-based insulin sensitivity is associated with a higher UACR measured at year-3 [19].

In the light of these publications, insulin sensitivity appears to be related with chronic renal disease in those with a compromised renal function. However, in our population of healthy people, this association was not apparent and none of the relations we observed were present in both sexes, and were not always concordant when the variables measuring renal function were analysed as continuous or as discrete variables. Sechi et al. showed that alterations of glucose metabolism in people with essential hypertension, are only evident for eGFR< 50 ml/min/1.73m^2^ and this may be the reason why our results are not conclusive [30].

Our results on UACR are unexpected, as a high baseline UACR, in comparison to an undetected level, was related with a higher insulin sensitivity three years later, and this was the case for men and women. UACR did increase over the three years of the study, as expected. While we have used UACR as a renal marker, it is also a marker of vascular function.

What are the possible mechanisms for an association between insulin sensitivity and chronic kidney disease? Low insulin sensitivity (as measured by the minimal model technique) has been described in people with renal disease but a normal eGFR (evaluated by inulin clearance); insulin sensitivity was similar across the range of eGFR [2]. These results imply that renal dysfunction, could precede the onset of declining insulin sensitivity. A rhesus monkey model provides additional arguments [31]. Recent studies have identified specific uremic toxins that could mediate an association between chronic renal disease and insulin sensitivity, toxins such as p-cresyl sulfate a protein in the intestinal microbiota [32].

In our healthy cohort, we showed that a higher filtration: eGFR (≥105 ml/min/1.73m^2^) was associated, cross-sectionally, with lower insulin sensitivity in women. This result is not so surprising as insulin resistance precedes the development of diabetes, which in turn is associated with a higher glomerular filtration rate [33]. However, the reverse was the case in men for our prospective study, as those with a higher eGFR were more likely to have a higher 3-year insulin sensitivity and a more pronounced increase in insulin sensitivity than the reference group, even if in the whole population both eGFR and insulin sensitivity decreased over time.

The multicentre aspect of this study is one of its strengths, as the study population covered a range of European lifestyles and diets. Differences between centres were accounted for in analyses by a random effect. At inclusion, insulin sensitivity was measured by the hyperinsulinemic-euglycemic clamp, a procedure that was carefully standardised across the European centres, for this large cohort study, with more than 1300 participants. All biological assays in the EGIR-RISC study are from central laboratories. At baseline the UACR was measured on two occasions and the mean used, leading to a more precise estimate. Another force is that there is little missing data in this study, and for the few variables where data were missing, we imputed with the sex-specific median value.

Our study differs from other studies in that all analyses have been done for men and women separately. This was justified by their differences in characteristics, and the study of interactions with sex. Other EGIR-RISC analyses have shown differences between men and women [20–22]. It is also unique in that we studied a cohort of healthy individuals, without chronic renal disease, diabetes, hypertension or dyslipidaemia.

The EGIR-RISC study has a number of limitations. The study population consists of healthy volunteers, and thus is not representative of the general healthy population of the same age. Further, as we excluded people who were not present at the three-year examination, we have selected an even healthier population, according to their characteristics at baseline. With the surrogate measures of insulin sensitivity, we were not able to precisely evaluate insulin sensitivity and its change over the three year follow-up period, even if the correlations with clamp based insulin sensitivity were of the order of 0.6. One of the major limitations of the EGIR-RISC study is that it is a very healthy population with only 15% of our population having an eGFR< 90 ml/min/1.73m^2^. There are likely to be only very small changes in parameters over three years in such a population, so a much longer follow-up would be required to show associations.

## Conclusion

Insulin sensitivity in the absence of chronic kidney disease (eGFR< 60 ml/min/1.73 m^2^) and without other markers of kidney damage, such as microalbuminuria, is not associated with a declining glomerular filtration rate. Our study is the only one, to our knowledge, that evaluates in a prospective study, insulin sensitivity as a function of baseline renal function. A longer prospective study evaluating insulin sensitivity by the reference method, both at baseline and at follow-up, over a range of eGFR values is needed to understand the physiopathology of change in insulin sensitivity in people with and without chronic kidney disease.

## Additional file

Additional file 1: **Figure S1.** Flow chart of the EGIR-RISC study: estimated glomerular filtration rate (eGFR), urinary albumin creatinine ratio (UACR). **Table S1.** Comparison of people included and not included in the analyses, median (quartile 1-quartile 3) and n (%). *P* values from Kruskal-Wallis and χ^2^ tests. The EGIR-RISC Study. **Table S2.** Spearman partial correlation coefficients, r_Sp,_ between the clamp measure of insulin sensitivity (M/I) and surrogate measures of insulin sensitivity, adjusted on age and recruitment centres, as fixed factors, by sex. **Table S3.** Differences (standard errors) in baseline insulin sensitivity indices (M/I, ISI and HOMA-IS) associated with one unit or class increase in baseline renal function parameters (estimated glomerular filtration rate (eGFR), urinary creatinine ratio (UACR)) from mixed models with fractional polynomial transformations where required (adjusted for age and for the recruitment centre as a random factor). The EGIR-RISC study. **Table S4.** Changes per year [median (quartile 1, quartile 3)] for continuous variables or n (%) for categorical variables between the 3-year follow-up and baseline, by sex. *P*-values from Kruskal Wallis or χ^2^ exact tests. The EGIR-RISC study. (DOCX 78 kb)

## Abbreviations

ALAT: Alanine aminotransferase
ASAT: Aspartate aminotransferase
BMI: Body mass index
CKD: Chronic kidney disease
CKD-EPI: Chronic Kidney Disease EPIdemiology collaboration equation
eGFR: Estimated glomerular filtration rate
EGIR-RISC: European Group for the study of Insulin Resistance - Relationship between Insulin Sensitivity and Cardiovascular disease
GGT: Gamma glutamyltransferase
HOMA-IR: Insulin resistance calculated from the HOmeostasis Model of Insulin Resistance
HOMA-IS: Insulin sensitivity calculated from 1/ HOMA-IR
IL-6: Interleukin-6
ISI: Matsuda insulin sensitivity index
M/I: Insulin sensitivity measured by clamp, glucose infusion/insulin concentration during the last 40 min of clamp
OGTT: Oral glucose tolerance test
UACR: Urinary albumin creatinine ratio
ΔHOMA-IS: Three year change in the HOMA-IS index
ΔISI: Three year change in the ISI insulin sensitivity index

## References

[1] DeFronzo RA, Alvestrand A, Smith D, Wahren J (1981). Insulin resistance in uremia. J Clin Invest.

[2] Fliser D, Pacini G, Engelleiter R, Kautzky-Willer A, Prager R, Franek E (1998). Insulin resistance and hyperinsulinémia are already present in patients with incipient renal disease. Kidney Int.

[3] Kubo M, Kiyohara Y, Kato I, Iwamoto H, Nakayama K, Hirakata H (1999). Effect of hyperinsulinemia on renal function in a general Japanese population: the Hisayama study. Kidney Int.

[4] Chen J, Muntner P, Hamm LL, Fonseca V, Batuman V, Whelton PK (2003). Insulin resistance and risk of chronic kidney disease in nondiabetic US adults. J Am Soc Nephrol.

[5] Onat A, Hergenç G, Uyarel H, Ozhan H, Esen AM, Karabulut A (2007). Association between mild renal dysfunction and insulin resistance or metabolic syndrome in a random nondiabetic population sample. Kidney Blood Press Res.

[6] Landau M, Kurella-Tamura M, Shlipak MG, Kanaya A, Strotmeyer E (2011). Health, aging and body composition study. Correlates of insulin resistance in older individuals with and without kidney disease. Nephrol Dial Transplant.

[7] Johns BR, Pao AC, Kim SH (2012). Metabolic syndrome, insulin resistance and kidney function in non-diabetic individuals. Nephrol Dial Transplant.

[8] Cheng HT, Huang JW, Chiang CK, Yen CJ, Hung KY, Wu KD (2012). Metabolic syndrome and insulin resistance as risk factors for development of chronic kidney disease and rapid decline in renal function in elderly. J Clin Endocrinol Metab.

[9] Pham H, Robinson-Cohen C, Biggs ML, Ix JH, Mukamal KJ, Fried LF (2012). Chronic kidney disease, insulin resistance, and incident diabetes in older adults. Clin J Am Soc Nephrol.

[10] Chen S, Chen Y, Liu X, Li M, Wu B, Li Y (2013). Association of insulin resistance with chronic kidney disease in non-diabetic subjects with normal weight. PLoS One.

[11] Park JH, Oh SW, Ahn SY, Kim S, Na KY, Chae DW (2013). Decreased estimated glomerular filtration rate is not directly related to increased insulin resistance. Diabetes Res Clin Pract.

[12] Jing C, Xu S, Ming J, Cai J, Zhang R, Shen H (2015). China National Diabetes and metabolic disorders study group: insulin resistance is not independently associated with chronic kidney disease in Chinese population: a population-based cross-sectional study. Clin Chim Acta.

[13] Matthews DR, Hosker JP, Rudenski AS, Naylor BA, Treacher DF, Turner RC (1985). Homeostasis model assessment: insulin resistance and beta-cell function from fasting plasma glucose and insulin concentrations in man. Diabetologia.

[14] Fox CS, Larson MG, Leip EP, Meigs JB, Wilson PW, Levy D (2005). Glycemic status and development of kidney disease: the Framingham heart study. Diabetes Care.

[15] Kurella M, Lo JC, Chertow GM (2005). Metabolic syndrome and the risk for chronic kidney disease among nondiabetic adults. J Am Soc Nephrol.

[16] Nerpin E, Risérus U, Ingelsson E, Sundström J, Jobs M, Larsson A (2008). Insulin sensitivity measured with euglycemic clamp is independently associated with glomerular filtration rate in a community-based cohort. Diabetes Care.

[17] Ryu S, Chang Y, Woo HY, Lee KB, Kim SG, Kim DI (2009). Time-dependent association between metabolic syndrome and risk of CKD in Korean men without hypertension or diabetes. Am J Kidney Dis.

[18] Hills SA, Balkau B, Coppack SW, Dekker JM, Mari A, Natali A, et al.; EGIR-RISC study Group The EGIR-RISC STUDY (the European group for the study of insulin resistance: relationship between insulin sensitivity and cardiovascular disease risk): I. Methodology and objectives. Diabetologia 2004;47:566–570. 10.1007/s00125-004-1335-5 14968294

[19] Pilz S, Rutters F, Nijpels G, Stehouwer CD, Højlund K, Nolan JJ, et al.; RISC Investigators Insulin sensitivity and albuminuria: the RISC study. Diabetes Care 2014;37:1597–1603. 10.2337/dc13-2573 24623021

[20] Kozakova M, Natali A, Dekker J, Beck-Nielsen H, Laakso M, Nilsson P, et al.; RISC Investigators Insulin sensitivity and carotid intima-media thickness: relationship between insulin sensitivity and cardiovascular risk study. Arterioscler Thromb Vasc Biol 2013;33:1409–1417. 10.1161/ATVBAHA.112.300948 23599442

[21] Petrie JR, Malik MO, Balkau B, Perry CG, Højlund K, Pataky Z, et al.; RISC Investigators Euglycemic clamp insulin sensitivity and longitudinal systolic blood pressure: role of sex. Hypertension 2013;62:404–409. 10.1161/HYPERTENSIONAHA.111.00439 23734006

[22] Rutters F, Besson H, Walker M, Mari A, Konrad T, Nilsson PM (2016). The association between sleep duration, insulin sensitivity, and β-cell function: the EGIR-RISC study. J Clin Endocrinol Metab.

[23] Levey AS, Stevens LA, Schmid CH, Zhang YL, Castro AF, Feldman HI (2009). CKD-EPI (chronic kidney disease epidemiology collaboration). A new equation to estimate glomerular filtration rate. Ann Intern Med.

[24] DeFronzo RA, Tobin JD, Andres R (1979). Glucose clamp technique: a method for quantifying insulin secretion and resistance. Am J Phys.

[25] Matsuda M, DeFronzo RA (1999). Insulin sensitivity indices obtained from oral glucose tolerance testing: comparison with the euglycemic insulin clamp. Diabetes Care.

[26] Royston P, Ambler G, Sauerbrei W (1999). The use of fractional polynomials to model continuous risk variables in epidemiology. Int J Epidemiol.

[27] Sauerbrei W, Meier-Hirmer C, Royston P (2006). Multivariate regression model building by using fractional polynomials: description of SAS, STATA and R programs. Comp Stat Data Anal.

[28] Boer IH, Mehrotra R (2014). Insulin resistance in chronic kidney disease: a step closer to effective evaluation and treatment. Kidney Int.

[29] Muniyappa R, Lee S, Chen H, Quon MJ (2008). Current approaches for assessing insulin sensitivity and resistance in vivo: advantages, limitations and appropriate usage. Am J Physiol Endocrinol Metab.

[30] Sechi LA, Catena C, Zingaro L, Melis A, De Marchi S (2002). Abnormalities of glucose metabolism in patients with early renal failure. Diabetes.

[31] Cusumano AM, Bodkin NL, Hansen BC, Iotti R, Owens J, Klotman PE (2002). Glomerular hypertrophy is associated with hyperinsulinemia and precedes overt diabetes in aging rhesus monkeys. Am J Kidney Dis.

[32] Koppe L, Pillon NJ, Vella R, Pelletier CC, Chambert S, Massy Z (2013). P-Cresyl sulfate promotes insulin resistance associated with CKD. J Am Soc Nephrol.

[33] Sun ZJ, Yang YC, Wu JS, Wang MC, Chang CJ, Lu FH (2016). Increased risk of glomerular hyperfiltration in subjects with impaired glucose tolerance and newly diagnosed diabetes. Nephrol Dial Transplant.

