# Supplementary material for: Renal function markers and insulin sensitivity after 3 years in a healthy cohort, the EGIR-RISC study
Source: BMC Nephrol. 2018 May 31;19:124. doi: 10.1186/s12882-018-0918-1 (PMC5984396; doi:10.1186/s12882-018-0918-1)
Supplement: Supplementary file 1 — Figure S1. Flow chart of the EGIR-RISC study: estimated glomerular filtration rate (eGFR), urinary albumin creatinine ratio (UACR). Table S1. Comparison of people included and not included in the analyses, median (quartile 1-quartile 3) and n (%). P values from Kruskal-Wallis and χ2 tests. The EGIR-RISC Study. Table S2. Spearman partial correlation coefficients, rSp, between the clamp measure of insulin sensitivity (M/I) and surrogate measures of insulin sensitivity, adjusted on age and recruitment centres, as fixed factors, by sex. Table S3. Differences (standard errors) in baseline insulin sensitivity indices (M/I, ISI and HOMA-IS) associated with one unit or class increase in baseline renal function parameters (estimated glomerular filtration rate (eGFR), urinary creatinine ratio (UACR)) from mixed models with fractional polynomial transformations where required (adjusted for age and for the recruitment centre as a random factor). The EGIR-RISC study. Table S4. Changes per year [median (quartile 1, quartile 3)] for continuous variables or n (%) for categorical variables between the 3-year follow-up and baseline, by sex. P-values from Kruskal Wallis or χ2 exact tests. The EGIR-RISC study. (DOCX 78 kb) [file 12882_2018_918_MOESM1_ESM.docx]

# Additional file 1

**Figure S1.** Flow chart of the EGIR-RISC study: estimated glomerular filtration rate (eGFR), urinary albumin creatinine ratio (UACR)

**1566** volunteers recruited

**247** not eligible for the EGIR-RISC study

**1319** participants selected

**60** participants not included as they had

hypertension, diabetes or dyslipidaemia

at baseline

**1259** participants included

in the EGIR-RISC cohort

**32** excluded as baseline data not

available for creatinine,

**933** participants studied

**405 men 528 women**

MEN: Analyses with eGFR and UACR WOMEN: Analyses with eGFR

|  | M/I | ISI | HOMA-IS |  |  | M/I | ISI | HOMA-IS |
| --- | --- | --- | --- | --- | --- | --- | --- | --- |
| Baseline | 405 | 375 | 393 |  | Baseline | 528 | 477 | 512 |
| Year 3 |  | 376 | 392 |  | Year 3 |  | 492 | 514 |
| Difference |  | 349 | 380 |  | Difference |  | 448 | 501 |

WOMEN: Analyses with UACR

|  |  |  |  | M/I | ISI | HOMA-IS |
| --- | --- | --- | --- | --- | --- | --- |
|  |  |  | Baseline | 520 | 469 | 504 |
|  |  |  | Year 3 |  | 486 | 507 |
|  |  |  | Difference |  | 442 | 494 |

**Table S1**. **Comparison of people included and not included in the analyses, median (quartile 1-quartile 3) and n (%). *P* values from Kruskal-Wallis and χ^2^ tests. The EGIR-RISC Study.**

| Variables | Population studied (n=933) | Population not studied  (n=326) | *P-value* |
| --- | --- | --- | --- |
| Age (years) | 44 (38-50) | 41 (35-48) | <.0001 |
| Women | 528 (57) | 177 (54) | 0.50 |
| Current smokers | 239 (26) | 94 (30) | 0.26 |
| Alcohol intake (g pure/week) | 50 (15-105) | 30 (0-81) | <.0001 |
| Physical activity (met-mins/week) | 2245  (1032-4746) | 2010  (792-4638) | 0.20 |
|  |  |  |  |
| BMI (kg/m^2^) | 24.7  (22.7-27.4) | 25.4  (22.6-28.6) | 0.047 |
|  |  |  |  |
| Systolic blood pressure (mmHg) | 118 (109-126) | 115 (108-124) | 0.02 |
| Heart rate (bpm) | 68 (60-75) | 68 (62-75) | 0.74 |
| **RENAL FUNCTION PARAMETERS** |  |  |  |
| Creatinine (μmol/l ) | 68 (58-79) | 65 (56-76) | 0.21 |
| eGFR (ml/min par 1.73 m^2^) | 106 (97-114) | 107 (101-111) | 0.34 |
| UACR (mg/mmol) | 0.21 (0-0.42) | 0.23 (0-0.43) | 0.34 |
| Microalbuminuria | 18 (2%) | 3 (1%) | 0.32 |
| **INSULIN SENSITIVITY INDICES** |  |  |  |
| M/I (µmol/min/Kg_ffm_/nM) | 133 (95-183) | 122 (85-171) | 0.006 |
| ISI | 9.3 (6.5-13.5) | 8.7 (5.5-13.1) | 0.033 |
| HOMA-IS | 15 (10-22) | 14 (9-22) | 0.052 |
| **BIOLOGICAL CHARACTERISTICS** |  |  |  |
| Fasting glucose (mmol/l) | 5.1 (4.8-5.4) | 5.0 (4.6-5.4) | 0.003 |
| 2h glucose (mmol/l) | 5.6 (4.7-6.6) | 5.6 (4.6-6.5) | 0.72 |
| Fasting insulin (pmol/l) | 30 (21-42) | 33 (21-50) | 0.008 |
| 2h insulin (pmol/l) | 143 (86-228) | 160 (90-270) | 0.046 |
|  |  |  |  |
| Total cholesterol (mmol/l) | 4.8 (4.3-5.4) | 4.6 (4.0-5.3) | 0.001 |
| LDL-cholesterol (mmol/l) | 2.9 (2.4-3.4) | 2.7 (2.2-3.4) | 0.022 |
| HDL-cholesterol (mmol/l) | 1.4 (1.2-1.7) | 1.4 (1.1-1.7) | 0.041 |
| Triglycerides (mmol/l) | 0.9 (0.7-1.3) | 0.9 (0.7-1.3) | 0.84 |
|  |  |  |  |
| Adiponectin (mg/l) | 8 (6-11) | 8 (5-10) | 0.35 |
| Leptin (ng/ml) | 10 (4-17) | 10 (5-20) | 0.18 |
| Aspartate aminotransferase (UI/l) | 20 (15-25) | 22 (18-27) | 0.0097 |
| Alanine aminotransferase (UI/l) | 13 (9-19) | 14 (10-21) | 0.24 |
| Gamma glutamyltransferase (UI/l) | 15 (10-21) | 13 (10-24 | 0.71 |
| Interlukin-6 (pg/ml) | 0.7 (0.5-1.2) | 0.7 (0.5-1.4) | 0.50 |
| 25-OH vitamine D (ng/ml) | 20 (13-27) | 14 (7-24) | 0.0074 |

**Table S2. Spearman partial correlation coefficients, r_Sp,_ between the clamp measure of insulin sensitivity (M/I) and surrogate measures of insulin sensitivity, adjusted on age and recruitment centres, as fixed factors, by sex.**

|  | Men | Women |
| --- | --- | --- |
| **Variables** | **r_Sp_** | **r_Sp_** |
|  |  |  |
| **Fasting samples** |  |  |
| HOMA-IS | 0.50 | 0.49 |
| Fasting insulin (pmol/l) | -0.53 | -0.50 |
|  |  |  |
| **Insulin and glucose sampled during OGTT** |  |  |
| Insulin sensitivity index (ISI) | 0.62 | 0.60 |
| 2 hour insulin (pmol/l) | -0.60 | -0.56 |
|  |  |  |

* *P*-values : all < .0001

**Table S3. Differences (standard errors) in baseline insulin sensitivity indices (M/I, ISI and HOMA-IS) associated with one unit or class increase in baseline renal function parameters (estimated glomerular filtration rate (eGFR), urinary creatinine ratio (UACR)) from mixed models with fractional polynomial transformations where required (adjusted for age and for the recruitment centre as a random factor). The EGIR-RISC study**

| Men | ln (M/I)  n=405 | | | ln (ISI)  n=375 | | | | | ln (HOMA-IS)  n=393 | | |
| --- | --- | --- | --- | --- | --- | --- | --- | --- | --- | --- | --- |
| Variables | difference  (SE) | *P*-value | difference  (SE) | | | *P* -value | | difference  (SE) | | *P* -value |  |
| Age (per 10 years) | -.001 (.027) | 0.52 | -.11 (.03) | | | <0.0005 | | -.076 (.032) | | 0.02 |  |
| Current smoker | -.083 (.052) | 0.11 | -.072 (.060) | | | 0.23 | | .024 (.061) | | 0.69 |  |
| Alcohol intake (per 100 g per week) | .023 (.024) | 0.35 | -.014 (.028) | | | 0.62 | | -.017 (.028) | | 0.53 |  |
| Physical activity (met-mins per week * 10^-5^) | 2.3 (.7) | 0.001 | 2.2 (.8) | | | 0.005 | | 1.8 (.8) | | 0.03 |  |
| Body mass index (kg/m^2^) | -.11 (0.01) ^i^ | <0.0005 | -.084 (.007) | | | <0.0005 | -.099 (.008) | | | <0.0005 |  |
|  |  |  |  | | |  | |  | |  |  |
| Systolic blood pressure (per 10 mmHg) | .0090 (.024) | 0.70 | -.030 (.026) | | | 0.25 | | -.026 (.027) | | 0.34 |  |
| Heart rate (per 10 bpm) | -.093 (.023) | <0.0005 | -.15 (.03) | | | <0.0005 | | -.14 (.03) | | <0.0005 |  |
|  |  |  |  | | |  | |  | |  |  |
| Cholesterol (mmol/l) | -.091 (.027) | 0.001 | -.13 (.03) | | | <0.0005 | | -.14 (.03) | | <0.0005 |  |
| LDL-cholesterol (mmol/l) | -.099 (.030) | 0.001 | -.14 (0.03) | | | <0.0005 | | -.14 (.04) | | <0.0005 |  |
| HDL-cholestérol (mmol/l) | .59 (.07) | <0.0005 | .62 (.08) | | | <0.0005 | | .55 (.09) | | <0.0005 |  |
| Ln(Triglycerides) (mmol/l) | -.41 (.04) | <0.0005 | -.46 (.00) | | | <0.0005 | | ^-^.45 (.05) | | <0.0005 |  |
|  |  |  |  | | |  | |  | |  |  |
| Adiponectin (mg/l) | .40 (.06) ^ii^ | <0.0005 | -.74 (0.11) ^iii^ | | | <0.0005 | | .053 (.010) | | <0.0005 |  |
| Leptin (ng/ml) | -.77 (.06) ^iv^ | <0.0005 | -1.0 (0.1) ^iv^ | | | <0.0005 | | ^v^ | | <0.0005 |  |
|  |  |  |  | | |  | |  | |  |  |
| Alanine aminotransferase (UI/l) | ^vi^ | <0.0005 | .55 (0.07)^vii^ | | | <0.0005 | | -.015 (.024) | | <0.0005 |  |
| Aspartate aminotransferase (UI/l) | -.0065 (.0029) | 0.03 | -.0086 (.0033) | | | 0.009 | | -.0061 (.0032) | | 0.06 |  |
| Gamma glutamyltransferase (UI/l) | ^viii^ | <0.0005 | ^xviii^ | | | <0.0005 | | ^ix^ | | <0.0005 |  |
|  |  |  |  | | |  | |  | |  |  |
| 1/IL-6 (pg/ml) | .12 (.03) ^x^ | <0.0005 | .12 (.04) ^x^ | | <0.0005 | | | .032 (.009) ^xi^ | | <0.0005 | |
| 25-OH vitamin D (ng/ml) | .0066 (.0024) | 0.006 | .0036 (.0027) | | | 0.19 | | .0020 (.0028)^x^ | | 0.47 |  |

The *P*-values shown above are from mixed models using fractional polynomials, adjusted for age and centre as random. The beta coefficients are only shown when there is one term in the fractional polynomial, transformations are shown by the superscripts.

i: (BMI/10)²

ii: ln (Adiponectin)

iii: √ (10/Adiponectin).

iv: √ (Leptin/10)

v: ln (Leptin), (ln (Leptin))^2^

vi: (ALAT/10)², (ALAT/10)² * ln (ALAT/10)

vii: √ (ALAT/10),

viii: GGT/100, (GGT/100) * ln (GGT/100)

ix: √ (GGT/100), GGT/100

x: 1/IL-6

xi: (1/IL-6)²

| Women | ln (M/I)  n=528 | | ln (ISI)  n=477 | | ln (HOMA-IS)  n=512 | | |
| --- | --- | --- | --- | --- | --- | --- | --- |
| Variables | difference  (SE) | *P*-value | difference  (SE) | *P*- value | difference  (SE) | | *P*-value |
| Age (per 10 years) | -.028 (.025) | 0.26 | -.11 (.03) | <.0005 | -.069 (.031) | | 0.03 |
| Current smoker | -.088 (.045) | 0.05 | .071 (.058) | 0.22 | .065 (.057) | | 0.26 |
| Alcohol intake (per 100g per week) | 5.4 (3.1) | 0.08 | 5.6 (3.9) | 0.15 | 6.4 (3.9) | | 0.10 |
| Physical activity (met-mins per week * 10^-5^) | 1.7 (.6) | 0.006 | 2.4 (.8) | 0.003 | 1.7 (.8) | | 0.04 |
|  |  |  |  |  |  | |  |
| Body mass index (Kg/m^2^) | -.041 (.005) | <.0005 | -.066 (.006) | <.0005 | -.071 (.005) | | <.0005 |
|  |  |  |  |  |  | |  |
| Systolic blood pressure (per 10 mmHg) | -.046 (.017) | 0.006 | -.095 (.021) | <.0005 | -.11 (.02) | | <.0005 |
| Heart rate (per 10 bpm) | -.061 (.019) | 0.002 | -.15 (.02) | <.0005 | -.16 (0.02) | | <.0005 |
|  |  |  |  |  |  | |  |
|  |  |  |  |  |  | |  |
| Cholesterol total(mmol/l) | -.037 (.025) | 0.14 | -.044 (.032) | 0.17 | -.041 (.032) | | 0.20 |
| LDL-cholesterol (mmol/l) | -.077 (.027) | 0.004 | -.11 (.03) | 0.001 | -.10 (.03) | | 0.003 |
| HDL-cholesterol (mmol/l) | .33 (.05) | <.0005 | ^i^ | <.0005 | ^i^ | | <.0005 |
| Triglycerides (mmol/l) | -.27 (.04) | <.0005 | -.41 (.05) | <.0005 | -.42 (.05) | | <.0005 |
|  |  |  |  |  |  | |  |
| Adiponectin (mg/l) | .039 (.005) | <.0005 | .58 (.06)^ii^ | <.0005 | .59 (.06)^ii^ | | <.0005 |
| Square root (Leptin) (ng/ml) | -.12 (.01) | <.0005 | -.21 (.01) | <.0005^4^ | -.22 (.01) | | <.0005 |
|  |  |  |  |  |  |  |  |
| Alanine aminotransferase (UI/l) | -.012 (.026) | <0.0005 | -.31 (.05)^iii^ | <.0005 |  | ^iv^ | <.0005 |
| Aspartate aminotransferase (UI/l) | -.0016 (.0027) | 0.55 | -.0052 (.0033) | 0.12 | -.0053 (.0034) | | 0.12 |
| Gamma glutamyltransferase (UI/l) | -0.13 (.03) | <.0005 | -.18 (.04) | <.0005 | -.16 (.04) | | <.0005 |
|  |  |  |  |  |  | |  |
| IL-6 (pg/ml) | .011 (.002)^vi^ | <.0005 | .17 (.02)^vii^ | <.0005 | .16 (.02)^vii^ | | <.0005 |
| 25-OH vitamin D (ng/ml) | .0027(.0018) | 0.12 | .0063 (.0022) | 0.004 | .0059 (.0022) | | 0.007 |

The *P*-values shown above are from mixed models, with the recruitment centre as random, using fractional polynomials. The beta coefficients are also shown when there is one term in the factional polynomial, with a linear model or a transformation as shown by the superscripts.

i: HDL^3^ +HDL^3^ln (HDL)

ii: ln (Adiponectin)

iii ln (ALAT)

iv: (ALAT /10)^2^ + (ALAT /10)^2^ * ln (ALAT /10)

v : ln (GGT)

vi: 10/ IL-6

vii: √ (10/ IL-6)

**Table S4. Changes per year [median (quartile 1, quartile 3)] for continuous variables or n (%) for categorical variables between the 3-year follow-up and baseline, by sex. *P*-values from Kruskal Wallis or χ² exact tests. The EGIR-RISC study.**

|  | Men | Women | *P*-value |
| --- | --- | --- | --- |
| **RENAL FUNCTION PARAMETERS** | |  |  |
|  | n=405 | n=528 |  |
| Creatinine (µmol/l) | 1.03 (-0.61, 2.75) | 0.92 (-0.86, 2.28) | 0.08 |
| eGFR (ml/min/1.73 m^2^) | -5.4 (-11.0, -0.7) | -5.0 (-10.7, 0.2) | 0.37 |
| eGFR classes |  |  |  |
| no change | 255 (63%) | 325 (62%) |  |
| worsened | 55 (14%) | 98 (18%) | 0.086 |
| improved | 95 (23%) | 105 (20%) |  |
|  |  |  |  |
|  | n=331 | n=441 |  |
| UACR (mg/mol) | 0.039 (-0.017, 0.089) | 0.049 (-0.023, 0.119) | 0.15 |
| UACR classes |  |  |  |
| no change | 130 (39%) | 172 (39%) |  |
| worsened | 144 (44%) | 202 (46%) | 0.70 |
| improved | 57 (17%) | 67 (15%) |  |
|  |  |  |  |
| **INSULIN SENSITIVITY INDICES** | |  |  |
|  | n=349 | n=448 |  |
| ISI | -0.49 (-2.48, 1.46) | -0.45 (-2.95, 1.94) | 0.73 |
|  | n=380 | n=501 |  |
| HOMA-IS | -0.92 (-5.18,3.07) | -0.74 (-5.29,3.85) | 0.53 |
